# Ancestral Patterning of Tergite Formation in a Centipede Suggests Derived Mode of Trunk Segmentation in Trilobites

**DOI:** 10.1371/journal.pone.0052623

**Published:** 2012-12-28

**Authors:** Javier Ortega-Hernández, Carlo Brena

**Affiliations:** 1 Department of Earth Sciences, University of Cambridge, Cambridge, United Kingdom; 2 Department of Zoology, University of Cambridge, Cambridge, United Kingdom; University of Otago, New Zealand

## Abstract

Trilobites have a rich and abundant fossil record, but little is known about the intrinsic mechanisms that orchestrate their body organization. To date, there is disagreement regarding the correspondence, or lack thereof, of the segmental units that constitute the trilobite trunk and their associated exoskeletal elements. The phylogenetic position of trilobites within total-group Euarthropoda, however, allows inferences about the underlying organization in these extinct taxa to be made, as some of the fundamental genetic processes for constructing the trunk segments are remarkably conserved among living arthropods. One example is the expression of the segment polarity gene *engrailed*, which at embryonic and early postembryonic stages is expressed in extant panarthropods (i.e. tardigrades, onychophorans, euarthropods) as transverse stripes that define the posteriormost region of each trunk segment. Due to its conservative morphology and allegedly primitive trunk tagmosis, we have utilized the centipede *Strigamia maritima* to study the correspondence between the expression of *engrailed* during late embryonic to postembryonic stages, and the development of the dorsal exoskeletal plates (i.e. tergites). The results corroborate the close correlation between the formation of the tergite borders and the dorsal expression of *engrailed*, and suggest that this association represents a symplesiomorphy within Euarthropoda. This correspondence between the genetic and phenetic levels enables making accurate inferences about the dorsoventral expression domains of *engrailed* in the trunk of exceptionally preserved trilobites and their close relatives, and is suggestive of the widespread occurrence of a distinct type of genetic segmental mismatch in these extinct arthropods. The metameric organization of the digestive tract in trilobites provides further support to this new interpretation. The wider evolutionary implications of these findings suggest the presence of a derived morphogenetic patterning mechanism responsible for the reiterated occurrence of different types of trunk dorsoventral segmental mismatch in several phylogenetically distant, extinct and extant, arthropod groups.

## Introduction

*“The question of (trilobite) thoracic segmentation is… whether the boundaries of the thoracic tergites coincide exactly with the (segment) boundaries or not… in the following I will avoid (this) difficult question…”*

J. Bergström, 1973.

**Figure 1:**
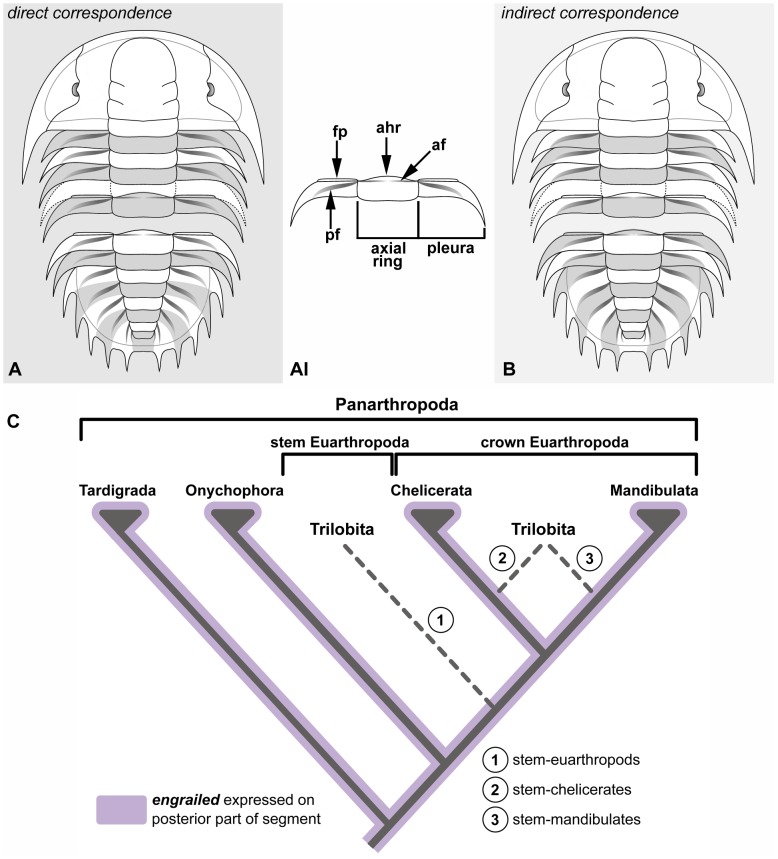
Opposing views on the interpretation of trilobite trunk segmentation and alternative phylogenetic hypotheses for the group. **A.** Direct correspondence between the segments and the dorsal exoskeletal plates (i.e. tergites) [11], [14]–[16], illustrated in the corynexochid *Olenoides serratus*; this interpretation is reflective of the plesiomorphic trunk segmentation present in most arthropods, in which the segment and tergite borders are coincident. Every second segment is shaded. **A1.** Morphological features and articulating devices of a typical trunk tergite, dorsal view. **B.** Indirect correspondence between segments and tergites [12], [19], [20]; the intersegmental boundaries of the trunk occur within each tergite, being delimited by the articulating (*af*) and pleural (*pf*) furrows. **C.** Trilobites have been variously interpreted as stem-euarthropods, stem-chelicerates, and stem-mandibulates [24], although most recent studies favour the latter affinities. The position of trilobites within total-group Euarthropoda indicates that the segment polarity gene *engrailed* had a role in the segmentation of these extinct organisms. Other abbreviations: *ahr*, articulating half ring; *fp*, fulcral process.

Trilobites comprise a very diverse and successful monophyletic group of well-known extinct arthropods characterized by the possession of a biomineralized dorsal exoskeleton, and include some of the oldest known macroscopic metazoans in the fossil record. All trilobites shared the same basic body construction; this consists of a head formed by (usually) four limb-bearing segments covered by a cephalic shield, followed by a homopodous trunk with a highly variable number of segments that show a significant diversity in terms of the number of expressed tergites (i.e. dorsal exoskeletal plates), as well as their degree of differentiation and fusion (i.e. tagmosis) [1] (Fig. 1). The clearly metameric organization of the trilobite trunk, coupled with the group’s rich fossil record, has sparked great interest in understanding the tempo and mode of segmentation in these extinct arthropods, and has produced considerable insights about their ontogenetic development and evolutionary trends [1]–[9].

### Matched or Mismatched Tergites?

There is a good understanding on the growth dynamics of the trunk in several trilobite species [10]; however, the precise details behind the underlying architecture of this tagma (i.e. specialized body region) are the subject of a polarizing debate. As recognized by various workers [1], [11], [12], [13], a controversial issue surrounding the interpretation of the segmental organization of the trilobite trunk relies on the uncertainty of whether the boundaries of the segmental units are reflected on the position of the dorsal exoskeletal elements. Either the borders of the tergites that constitute the trilobite trunk are aligned with the underlying intersegmental borders in a one-to-one correspondence [11], [14]–[16] (Fig. 1A), as is the case in most arthropods [17], [18], or they are not [12], [19], [20]. The latter scenario proposes that the intersegmental borders of the trunk segments are out of phase by approximately half a tergite, and thus are aligned with the articulating and pleural furrows (Fig. 1B). Størmer [12] pioneered this interpretation based on observations of the posterior morphology of the cephalic shield in some Cambrian olenellid trilobites. More importantly, however, Størmer [12] also relied extensively on extrapolations from the traditional concept of ‘secondary segmentation’ developed by Snodgrass [21], which proposes that the segmental organization of the longitudinal musculature in adult arthropods is reflective of the ‘primary segmentation’ (i.e. myosegmentation) pattern inherited from an annelid ancestor. A few other studies have further developed Størmer’s hypothesis based on detailed examinations of trilobite post-cephalic morphology, inferred sites of muscle attachment [20], and the early ontogenetic growth of some species [19]. Despite these supporting data, the core principle of Størmer’s trunk segmentation model has been challenged on the grounds that trilobites are too primitive to display such complex internal trunk arrangement [14], or over a general disagreement with the interpretation of the significance of the various exoskeletal features (e.g. glabellar and pleural furrows) [11], [15], [16].

The main arguments utilized by Størmer [12], and later adopted by Hessler [20], rest on the assumption that the generalized arthropod trunk is reflective of a secondarily derived mode of segmentation [21], an interpretation that has been proven erroneous with the implementation of molecular biology techniques that allow the expression of different genes and proteins through embryonic development to be traced [22]. Certainly, it is now recognized that arthropods are not as closely related to annelids as once thought (i.e. Articulata hypothesis), but rather belong to a group of metazoans characterized by the shedding of their chitinous exoskeletal cuticle during growth (i.e. Ecdysozoa hypothesis) [23], [24]. It is noteworthy that, although all previous studies that have actively tackled the problem of the trilobite trunk segmentation were performed after the first reports of trilobites with soft tissue preservation [1], most of them were published before the detailed investigations of specimens with exceptionally preserved ventral structures became available [1], [13], [25]–[32]. The general understanding of the organization of the trilobite trunk has advanced considerably in the last decades, and thus it is now possible to address this problem within the context of current arthropod palaeobiology and developmental biology.

Clarifying the correspondence between the tergites and the segments that constitute the trilobite trunk carries implications for understanding the origins and early evolutionary history of this important group, as it has been suggested that certain intrinsic aspects of trilobite trunk development show indications of significant variability and plasticity, particularly evident in Cambrian representatives relative to younger forms [2], [34]–[36]. Other studies have proposed that the plasticity of the trilobite trunk can be attributed to external ecological factors, rather than to a “loose” internal control of the development [6]. Regardless of the cause, the potential recognition of segmental mismatch in the trilobite trunk [12], [19], [20] is suggestive of a considerable, and largely unexplored, degree of developmental complexity in some of the earliest arthropods in the fossil record, and could have a direct impact on the studies that focus on aspects of the group’s functional morphology [11], [27], [32], [33], [36].

### *engrailed* and Tergite Formation

The loss of biological information associated with the process of fossilization makes it impossible to examine directly the relationship of the trilobite’s tergites and their corresponding segmental units. However, several of the mechanisms responsible for the formation and patterning of the segments in extant arthropods are highly conserved, and thus enable making some inferences about the fundamental genetic processes required for the construction of a metameric trunk in these extinct representatives. A prime example can be found in the segment polarity gene *engrailed* (*en*), which plays a pivotal role during segmentation, as it is essential for the formation and maintenance of the intersegmental borders in *Drosophila* [38]–[40], and at embryonic and postembryonic stages is expressed as transverse stripes that define the posterior region of each segment in all the representatives of Panarthropoda (Tardigrada+Onychophora+Euarthropoda) in which it has been examined [18], [41]–[45]. Although the precise phylogenetic position of trilobites has been controversial throughout the years, their affinities lie firmly within total-group Euarthropoda [17], [24], and thus *en* was in all likelihood also involved in segment formation in these extinct arthropods [46] (Fig. 1C).

Some studies have also found a direct correlation between the expression of *en* and the position of the tergite boundaries in the larval and adult abdomen of insects such as *Oncopeltus fasciatus* and *Drosophila melanogaster* [41], [47]–[50] as well as the embryonic trunk of the diplopod *Glomeris marginata* [43], [51]. These observations are particularly important, as they strongly suggest that it is possible to infer, albeit tentatively, the expression domain of *en* based on the position of the dorsal exoskeletal elements, even in cases in which there is a secondary modification on the exact expression domain of the *en* stripe, such as the dorsal side of *Glomeris* [43], [51] (see discussion below). However, insects and diplopods have derived types of trunk segmentation and development relative to the primitive arthropod condition [17], [24], [52], [53], and thus it is uncertain whether the association between the expression of *en* and the formation of the tergite borders in these arthropods is representative of the ancestral state. To address this question, it is necessary to analyze the correspondence between *en* and the tergites in another extant arthropod model, ideally one that features a plesiomorphic trunk morphology.

### *Strigamia maritima* as Model for Tergite Formation

Due to the conservative trunk morphology of centipedes, most notably the homonomy of the segments and direct correspondence between dorsal and ventral sclerites, we utilize the geophilomorph *Strigamia maritima* to study the correspondence between the formation of the tergites, during late embryonic to postembryonic development, and the dorsoventral expression of *en*. We then use the information on the correlation of *en* expression and tergite border formation as the theoretical foundation from which to make inferences about the expression domains of this segment polarity gene in the trunk of exceptionally well-preserved trilobites, and other closely related fossil arthropods. The trunk segments of *Strigamia* have several desirable traits for the aims of this study, such as the anteroposterior differentiation of the tergite into a pretergite and a metatergite, and the presence of lateral spiracles, as these morphological features can be utilized to follow the development of the tergites. Furthermore, the embryonic development of *Strigamia* has recently been described in detail [54], and thus it is now possible to follow the precise timing of tergite formation in the late embryonic stages and through early postembryonic instars.

## Materials and Methods

### Embryo Collection, Culturing, Staging and Fixation

Egg clutches of *Strigamia* were collected near Brora, northeastern Scotland (see [54] for locality details). Some of them were collected and fixed in the field (summer 2007), other were collected alive in June 2011, transported and later cultured in the lab as described by Brena and Akam [54]. Collection of live specimens in the field did not require any specific permits as the site is not privately owned nor protected, and there was no interaction with endangered or protected wildlife. At the lab the eggs were regularly checked under the dissecting microscope for new hatchlings; these were allowed to develop until the desired postembryonic stage (CB, unpublished data) then fixed in 4% formaldehyde in phosphate-buffered saline (PBS). Specimens were thoroughly washed in PBT (PBS with 0.1% Tween-20) and dehydrated in a graded series of methanol (30%, 50%, 70%) and stored in 100% methanol at −20°C. Staging of embryonic stages follows that described previously [54].

### Dissection

Prior to dissection, the embryos were rehydrated in a methanol:PBT series to 100% PBT. Pre-hatchling individuals were dechorionated manually. In all individuals, embryonic and postembryonic exuviae were removed using a pair of fine tweezers (Dumont 55). As noted by several workers [44], [54], [55], the deposition of the cuticle during all the developmental stages analysed in this study can represent an obstacle that hinders the direct interaction of the RNA probe with the target tissues and/or produce a non-specific staining. Consequently, specimens were dissected using tweezers, a fine scalpel and a delicate brush to carefully extract the gut/yolk in order to facilitate access of the RNA probe to the visceral side of the body wall.

### *engrailed* Staining and Visualization

In situ hybridizations were mostly performed as described by Chipman et al. [56]; the only difference consisted in the utilization of a lower concentration of anti-DIG antibody (1∶2000–3000 dilution in 10% goat serum/PBT) during incubation. After staining the probe for visualization, all the material was nuclear counterstained with Sytox Green (0.1% in PBT) for 1 hour at room temperature, then transferred gradually to 70% glycerol and stored in 9-well plates at 4°C. Stained material was photographed as whole mounts and flat mounts as described by Brena and Akam [54]. Due to the size and natural curvature of the embryos [54], [55], most individuals were further dissected to allow flat mounting with minimal overlapping of the dorsoventral tissues.

### Fossil Material

Information on the morphology of the exoskeleton in trilobites and trilobite-like taxa was extracted from the primary literature, original photographs, and/or by direct inspection of catalogued specimens housed in scientific collections. The Smithsonian Institution (Washington D. C.), the Palaeontological Association (UK), and the Whittington Archives (University of Cambridge, UK) granted permission for the study of collections and figure reproduction. Institutional abbreviations: Smithsonian Institution (USNM); National Museum of Wales (NMW); Early Life Research Centre, Nanjing Institute of Geology and Palaeontology, China (ELRC). No new material was collected for this study.

## Results

### *engrailed* Expression, Tergite Formation and Dorsal Closure in Late Embryonic Stages

#### Embryonic stage 7

**Figure 2:**
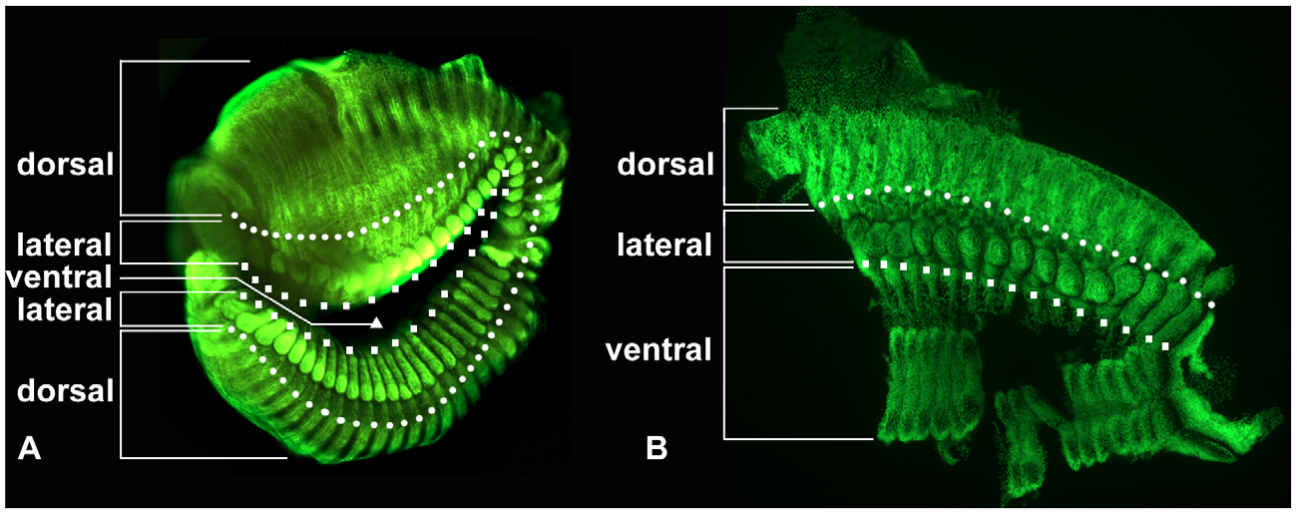
Major body components in stage 7–8 embryos of *Strigamia maritima.* Specimens photographed with fluorescent nuclear staining (Sytox Green) to show the morphology. **A.** Lateral view of whole-mount embryo corresponding to Fig. 4D, head on the upper left. The juxtaposed ventral surface (*arrow*) of the embryo is obscured and out of sight. **B.** Flat-mounted posterior region of embryo corresponding to Fig. 2C, anterior to the left. In both A and B dots indicate the approximate boundary between the prospective dorsal and lateral tissues as observed in the adult. Squares indicate the approximate boundary between the prospective lateral and ventral tissues as observed in the adult.

**Figure 3:**
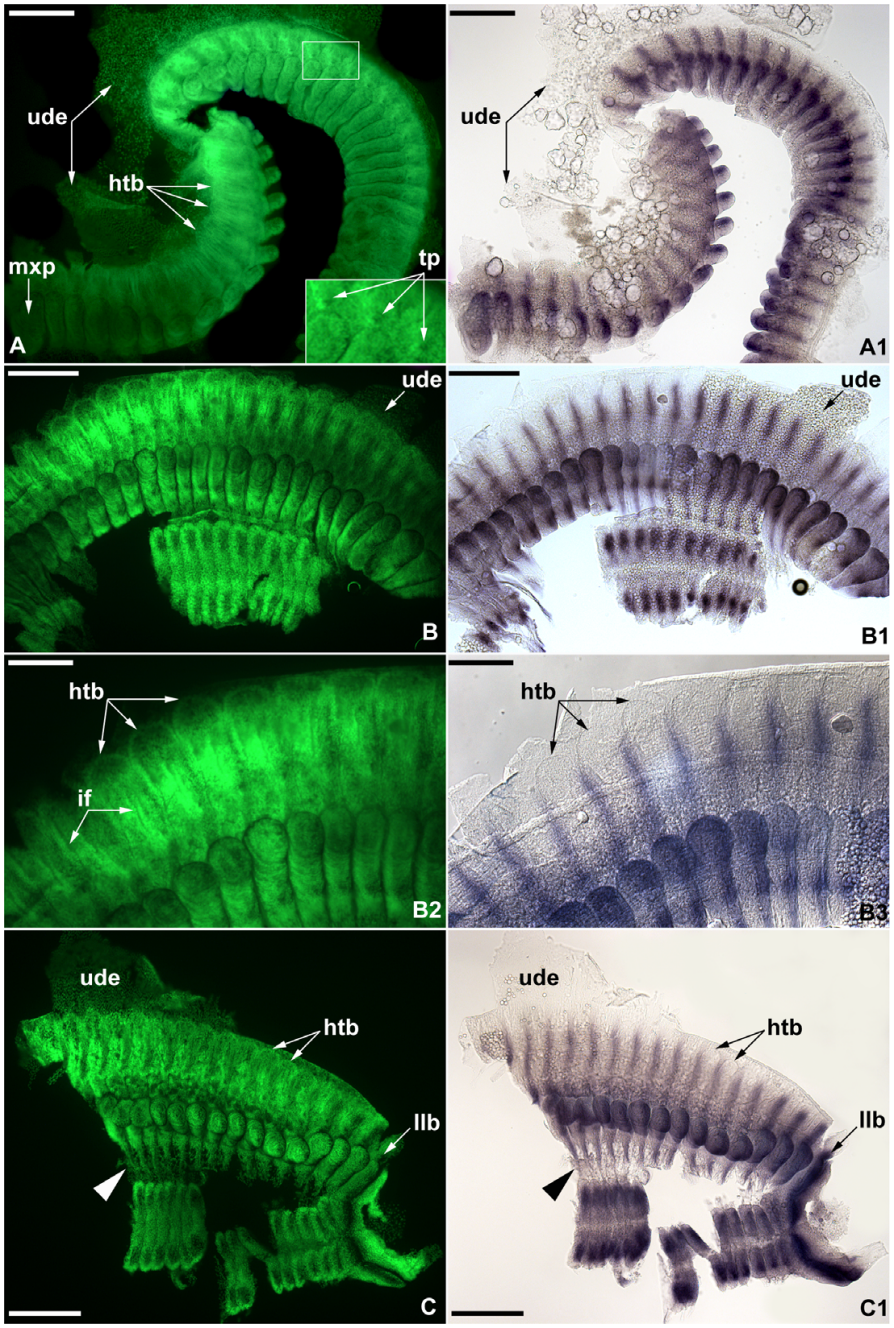
Hemitergite development and *engrailed* expression in flat mounted stage 7 embryos of *Strigamia maritima*. All images orientated with anterior to the left. Images with the same letter indicate same specimen; A, B, B2, C: fluorescent nuclear staining (Sytox Green) to show the morphology; A1, B1, B3 and C2 respectively same view as A, B, B2, C, under transmitted light, showing pattern of *en* expression. **A–A1.** Lateral view of most of the left half of a germ band, showing developing hemitergites extended along the dorsal side only for 30% in the anterior half of the trunk, and the presence of developing tracheal pits (*tp*). For most of the germ band only the laterodorsal part is visible; note the lack of gene expression in the undifferentiated dorsal epithelium (*ude*). **B–B1.** Ventral view of middle trunk region. **B2–B3.** Close-up of panel B, showing developing hemitergites with weakly formed intercalary furrow (*if*). **B3.** Shows correspondence between the extent of the laterodorsal *engrailed* stripe and the hemitergite extension. **C–C1.** Ventral view of posterior trunk of nuclear stained embryo. At this stage a thin undifferentiated epithelium, not expressing *engrailed*, is interposed between the medial and lateral parts of the germ band (*arrowhead*). **C1.** Shows posterior correspondence between *engrailed* expression and hemitergite coverage. The last leg-bearing segment (*llb*) features the only dorsoventrally uniform *engrailed* stripe. Other abbreviations: *htb,* semicircular dorsal border of the hemitergites; *mxp*, maxilliped. Scale bars: 200 µm for A, A1, B, B1, D, D1; 100 µm for insert in A, C, C1.

The *Strigamia* embryo develops as an extended flat germ band on the surface of the egg. In this flattened germ band, the ectoderm is on the surface, with the ventral component along the medial area and the right and left laterodorsal components symmetrically at the two sides [54] (Fig. 2). During embryonic stage 6 this germ band ‘sinks’ into the yolk (i.e. dorsoventral flexure), bending approximately half way and conferring to the embryo a distinctly bent appearance (Fig. 2A). Consequently, from stage 7 onwards the head of the resulting *Strigamia* embryo faces the proctodeum, and the anterior part of the ventral ectoderm juxtaposes with the posterior part (shown in Fig. 2B). At the lateral edges of the juxtaposition area, the two symmetrical lateral tissues, including the limb buds, tend to juxtapose in the same way (Fig. 3A). The two halves of the dorsal tissue, constituted by the series of the forming right and left halves of the developing tergites (i.e. hemitergites), still in a lateral position at the beginning of stage 7 will then extend - and eventually meet - dorsally (i.e. dorsal closure) (Fig. 2A).

The pattern of *en* from an original uniform transversal stripe at the posterior edge of each segment during early germ band stages [56] is differentiated along the mediolateral axis into domains of higher and lower level of expression, associated with the differentiation of the various morphological structures, including the limb buds (Fig. 3). On the laterodorsal sides of the embryo, the expression domains consist of distinctly continuous stripes that are restricted to the posterior portion of each segment, and which continue into the posterior margin of the corresponding limb buds (Figs 3A1, B1, C1). The dorsal extension of each transverse *en* stripe in this domain follows very closely that of the developing hemitergite, as the latter extends dorsally to eventually fuse with the corresponding hemitergite on the other side of the body, forming a full tergite at the moment of dorsal closure. The correlation between the extension of *en* stripe and hemitergite is maintained throughout the whole length of the trunk, with the semicircular dorsal border of each hemitergite being only slightly longer than the expression stripe (Fig. 3B3). At this stage, the transverse width of each hemitergite, and its expression stripe, covers approximately 20–30% of each laterodorsal side of the anterior region of the embryo (Fig. 3A); on the posterior segments, the coverage is approximately 50% due to the fact that most of the yolk mass is confined anteriorly (Figs 3B–C1). There are no signs of gene expression in the associated undifferentiated dorsal epithelium that engulfs the embryo prior to complete closure (Figs 3A, B2, B3, C).

**Figure 4:**
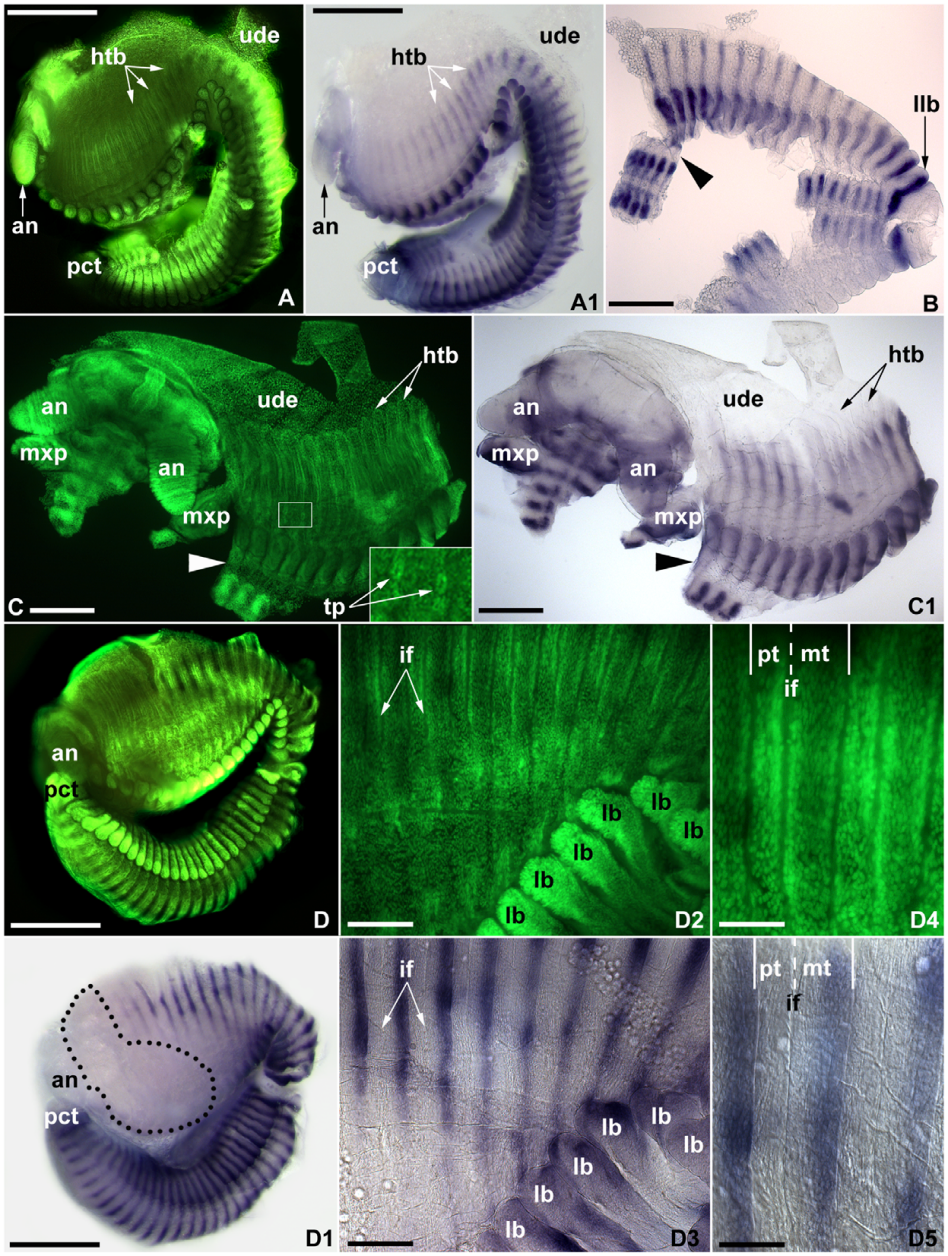
Hemitergite development and *engrailed* expression in whole mount (A, C, C3) and flat mounted (all others) stage 8 embryos of *Strigamia maritima*. All images orientated with anterior to the left. Images with the same letter indicate same specimen; A, C, D, D2 and D4: fluorescent nuclear staining (Sytox Green) to show the morphology; A1, C1, D1, D3 and D5 respectively same view as A, C, D, D2 and D4, under transmitted light, showing pattern of *en* expression. **A–A1.** Lateral view of whole-mount embryo with approximately 60% hemitergite extension on the anterior region of the trunk; note the correlation between the hemitergite dorsal border (*htb*) and the extent of the *engrailed* stripe. **B.** Ventral view of posterior trunk of embryo under transmitted light; note the reduced continuous dorsoventral *engrailed* stripe corresponding to the last leg-bearing segment (*llb*) at the posterior end. **C–C1.** Oblique view of embryo showing anterior region with approximately 75% of hemitergite dorsal extension and undifferentiated dorsal epithelium (*ude*). The tracheal pits (*tp*) become more conspicuous at this stage (insert). **D–D1.** Lateral view of whole-mount embryo with 90% hemitergite extension on the anterior region; note the correlation with the extent of the *engrailed* expression stripe. The anteriormost part of the trunk lacks expression due to the presence of residual yolk during dissection (*dotted line*). **D2–D3.** Close up of developing hemitergites and limb buds (*lb*). The morphology of the intercalary furrow (*if*) has become more accentuated. **D4–D5.** Detail of hemitergites, showing the anterior and posterior tergite borders (*solid lines*) and the intercalary furrow (*dashed line*), and together creating a clear distinction between the pretergite (*pt*) and metatergite (*mt*); note that the *engrailed* expression stripe is confined to the posterior portion of the (meta)tergite. Other abbreviations: *an*, antennae; *mxp*, maxilliped; *pct,* proctodeum; *arrowhead*, undifferentiated epithelium. Scale bars: 400 µm for A, A1, D, D1; 200 µm for B, C, C1; 100 µm for D2, D3; 50 µm for D4, D5; 75 µm for insert in C.

Although the hemitergites have a simple petal-like appearance at this stage, a number of morphological features become apparent. The most conspicuous is the presence of the intercalary furrow (Fig. 3B2) [54] that superficially separates the hemitergite into the pretergite-metatergite configuration observed in adult geophilomorphs [57]. It is also possible to observe the presence of tracheal pits (i.e. ectodermal invaginations that will eventually form the spiracles after full development) confined to the posterior half of the hemitergite (Fig. 3A) [54]. The ventral highly expressed *en* domains are restricted to a pair of short bands within the posterior half of each segment, possibly associated with the formation of the ventral exoskeletal plates (i.e. sternites) (Figs 3B1, C1). On the posteriormost leg-bearing segment, however, there is a notable exception, as the *en* stripe is strongly expressed in almost perfect continuity from the ventral midline to the laterodorsal tissues (Fig. 3C1). This stripe probably represents the early stages of molecular determination associated with the formation of the last leg-bearing segment, only appearing clearly morphologically by stage 8 (Fig. 4B) [54]. A weak staining was detected in the fine epithelium underlying the limb buds that connects the medioventral and lateral parts of the germ band (Fig. 3C).

#### Embryonic stage 8

In the last stage prior to hatching, the expression of *en* is very similar to that previously described for stage 7 embryos, including the correlation between the laterodorsal extent of the developing hemitergite and the corresponding stripe (Figs 4A, C, D). At this point, the transverse extension over the dorsal area of the anterior hemitergites has increased to approximately 60–90% (Figs 4A, C, D), whilst the posterior hemitergites have almost completed dorsal closure (Figs 4D, D1). The total area of the undifferentiated dorsal epithelium has become reduced due to the extension of the hemitergites (Fig. 4C). Stage 8 represents the onset of the embryonic apolysis (i.e. separation of the cuticle from the epidermis during the moulting process) [54], and thus it is possible to observe loose portions of the exuvia in some of the limbs, particularly the antennae (Fig. 4C1). The overall morphology of the hemitergites remains fairly simple, albeit considerably more elongated than in stage 7. The intercalary furrow, however, has become more defined, and it is possible to observe the tergite borders with clarity (Figs 4C, D2–D5). Without the reference provided by the *en* stripe, it is difficult to discern between the tergite borders and the intercalary furrow at high magnifications, both under light and fluorescent microscopy, as the difference in width between these structures is very subtle. The total width of the tergite can be divided into 40% pretergite and 60% metatergite at this stage. The position of the *en* stripe indicates that the differentiation between pretergite and metatergite represents a specialization of the cuticle within the same segment, not depending on reiterated *en* expression. The presence of the developing tracheal pits also becomes more accentuated (Fig. 4C). The medioventral expression domains remain essentially the same as in the previous stage. The continuous posteriormost stripe defines the posterior border of the last leg-bearing segment, by now morphologically defined (Fig. 4B) [54].

### Dorsal and Lateral Differentiation of Sclerites in Post-embryonic Stages

#### *Engrailed* expression in early juveniles

All attempts to obtain a tissue-specific signal in postembryonic individuals were unsuccessful. In most cases, the staining pattern consisted of isolated patches throughout the specimen, in association with dissection sites, or non-specific accumulation in the limbs. The practical difficulties associated with performing in situ hybridization and staining in heavily sclerotized tissues can be attributed to the physical obstruction of the RNA probe due to the presence of impermeable biological components, even in the visceral side of the body wall. The possible significance of these observations is dealt with in the discussion. The identification of early post-embryonic stages is the result of an independent analysis (CB, unpublished data).

#### Post-hatching stages

**Figure 5:**
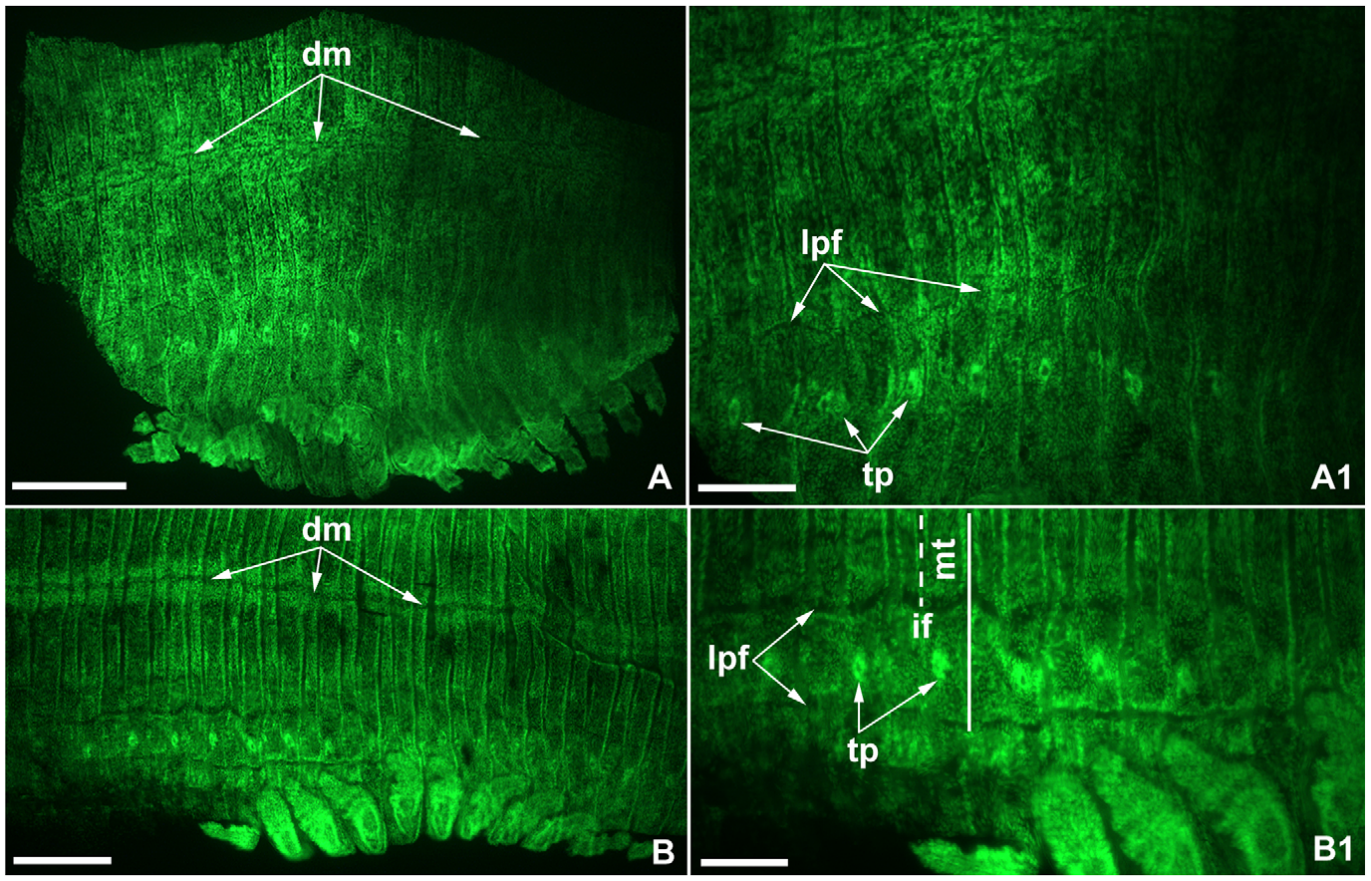
Laterodorsal sclerite differentiation in flat mounted post-hatching juveniles of *Strigamia maritima*. All images orientated with anterior to the left. Images with the same letter indicate same specimen. All specimens photographed with fluorescent nuclear staining (Sytox Green) to show the morphology. **A.** Laterodorsal view of juvenile showing anterior trunk region and completed dorsal closure indicated by dorsal midline (*dm*). **A1.** Close up showing incipient formation of the longitudinal pleural furrows (*lpf*) in the lateral region. **B.** Laterodorsal view of juvenile, showing middle trunk region. **B1.** Close up showing similar morphological features as A1 for the middle of the trunk. The intersection between the longitudinal furrows and the tergite borders (*solid line*) form quadrants that will become distinct sclerites. The position of the intercalary furrow (*if*/*dashed line*) indicates that the presumptive tracheal pits (*tp*) are aligned with the metatergite (*mt*). Scale bars: 200 µm for A, B; 100 µm for A1, B1.

Prominent changes observed in the cuticle of post-hatchling juveniles include the completed dorsal closure, forming a dorsal midline running longitudinally throughout the trunk (Figs 5A, B), as well as the first signs of differentiation of the lateral sclerites (Figs 5A1, B1). Since the hemitergite borders have met dorsally, there are no residual signs of the undifferentiated epithelium (e.g. Fig. 4C). The intercalary furrow deepens further, and it is possible to observe a stronger differentiation between the smaller pretergite and the larger metatergite, with the former representing approximately 30% of the total width of the whole tergite (Fig. 5B1). The most notable new feature is the appearance of a pair of parallel, longitudinal furrows on each side that represent the early stages of differentiation of the pleural region (Figs 5A1, B1). These longitudinal pleural furrows are perpendicular to the intercalary furrow and the tergite borders, and run dorsally and ventrally relative to the tracheal pits, which are dorsoventrally aligned with the metatergite (Fig. 5B1). Based on the position of the tracheal pits, the dorsal longitudinal pleural furrow defines the prospective lateral border of each tergite, whilst the ventral longitudinal pleural furrow demarcates the junction with the sclerites surrounding the prospective coxal armature. The superimposition of the tergite borders and the intercalary furrow with the dorsal longitudinal pleural furrows results in a number of repetitive pleural quadrants (Figs 5A1, B1).

#### Peripatoid

**Figure 6:**
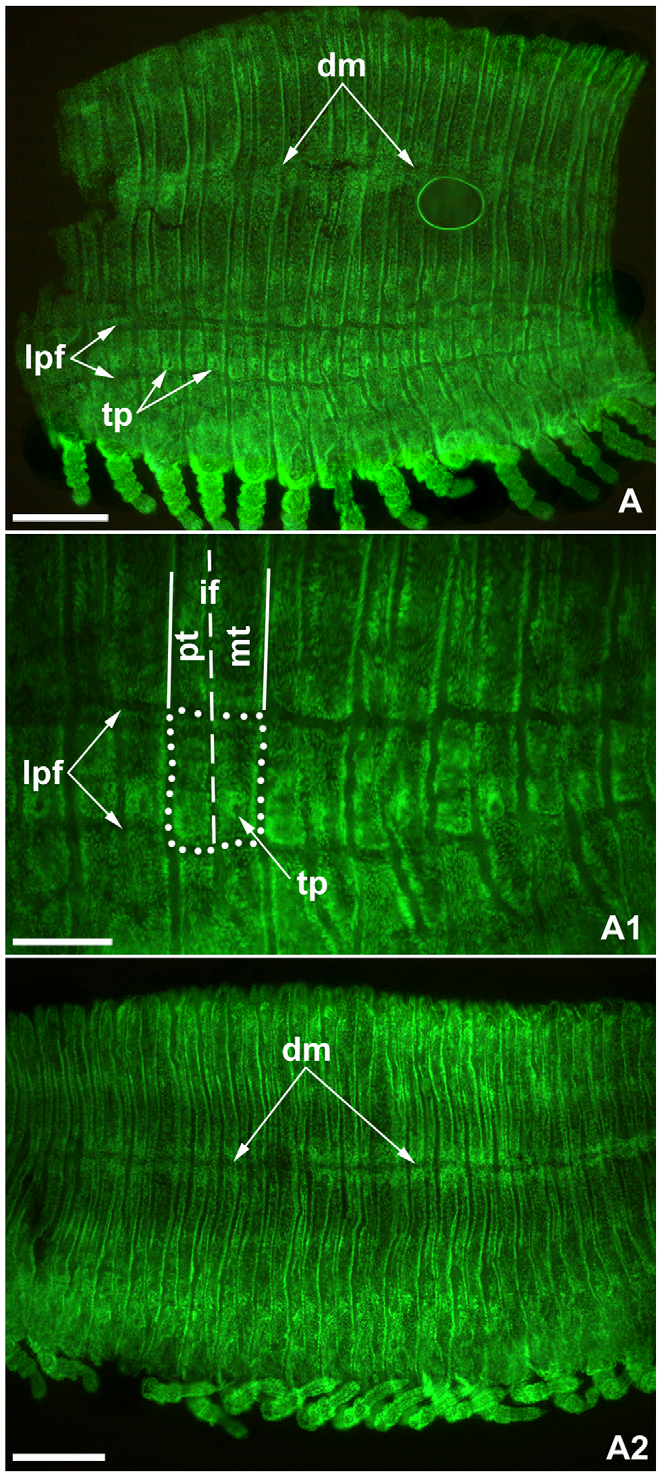
Laterodorsal sclerite differentiation in flat mounted peripatoid juveniles of *Strigamia maritima*. All images orientated with anterior to the left. Images with the same letter indicate same specimen. All specimens photographed with fluorescent nuclear staining (Sytox Green) to show the morphology. **A.** Laterodorsal view of juvenile, showing anterior trunk region with dorsal midline (*dm*) and well-developed longitudinal pleural furrows (*lpf*) and presumptive tracheal pits (*tp*). **A1.** Close-up of pleural region, showing pleural quadrants (*dotted line*) formed by the intersection of the tergite borders (*solid lines*), the intercalary furrow (*if/dashed line*) and the longitudinal pleural furrows. **B.** Laterodorsal view of same specimen, showing middle trunk region. Other abbreviations: *pt*, pretergite; *mt*, metatergite. Scale bars: 200 µm for A, A2; 100 µm for A1.

Most of the features described before become more accentuated (Fig. 6). The lateral borders of the tergites (i.e. dorsal longitudinal furrow, Figs 6A, A1) are further differentiated, and approximate the mature morphology, although it is still possible to observe strong traces of the dorsal midline (Figs 6A, A2). The previously faint longitudinal pleural furrows have become very well defined, and there are some signs of differentiation around the site of limb attachment (Fig. 6A1). Each pleural quadrant has become further subdivided by a medial transverse furrow that is in continuity with the intercalary furrow of the tergites, which separates the tracheal pit-bearing posterior anlagen from the still featureless anterior one (Fig. 6A1); this marks a more advanced stage in the differentiation of the pleural sclerites. It is possible to observe different degrees of pleural differentiation along the anteroposterior axis of the trunk (Figs 6A, A2); however, there is some variability within individuals in this regard.

#### Foetus

**Figure 7:**
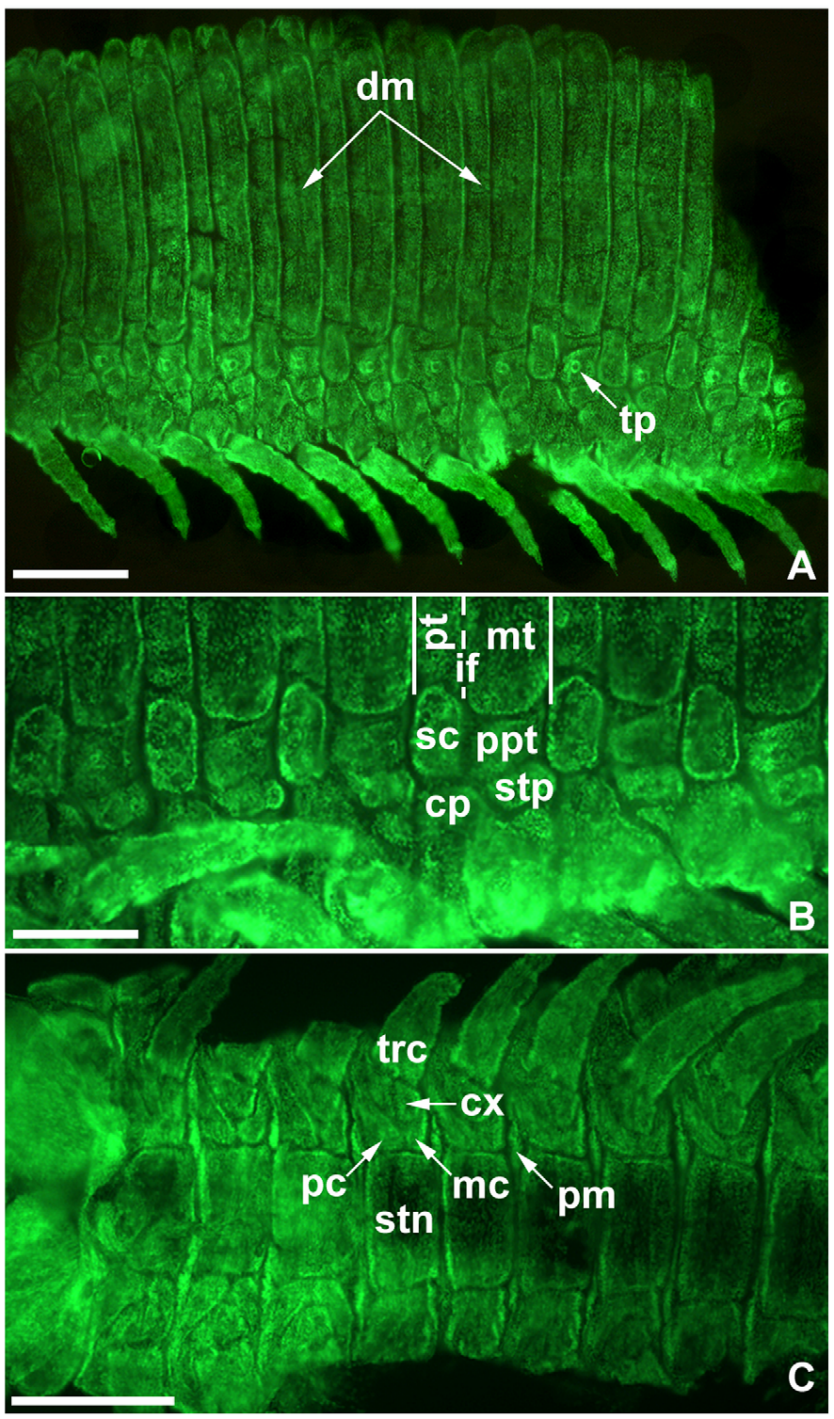
Laterodorsal sclerite differentiation in flat mounted foetus juveniles of *Strigamia maritima*. All images orientated with anterior to the left. Images with the same letter indicate same specimen. All specimens photographed with fluorescent nuclear staining (Sytox Green) to show the morphology. **A.** Laterodorsal view showing middle trunk region and differentiated pleural sclerites. **B.** Lateral view of middle trunk region, showing detail of the differentiated eupleurium (i.e. lateral sclerites of a leg-bearing segment [56]) **C.** Ventral view showing anterior trunk region. Abbreviations: *pt*, pretergite; *mt*, metatergite; *ppt*, principal paratergite; *sc*, scutellum; *stp*, stigmatopleurite; *cp*, catapleurite; *pc*, procoxa; *mc*, metacoxa; *stn*, sternite; *cx*, coxa; *trc*, trochanter; *pm*, interpleural membrane. Scale bars: 200 µm for A, C; 100 µm for B.

All the exoskeletal elements have acquired, or are close to, the mature morphology (Fig. 7). The pretergite and metatergite are clearly differentiated, and their proportions are reflective of the adult phenotype. The dorsal midline has become very faint. The pleural quadrants have differentiated into the different sclerites that conform the adult eupleurium [57], including the stigmatopleurite (i.e. sclerite bearing the tracheal pit), scutellum, principal paratergite, and catapleurite (Figs 7A, B). The ventral sclerites have also become completely differentiated, and it is possible to observe traces of the interpleural membrane between the sternites, as well as the exoskeletal armarture around the limb consisting of the procoxa and metacoxa (Figs 7B, C).

## Discussion

### Dorsal *engrailed* Expression and Tergite Formation in Euarthropoda

**Figure 8:**
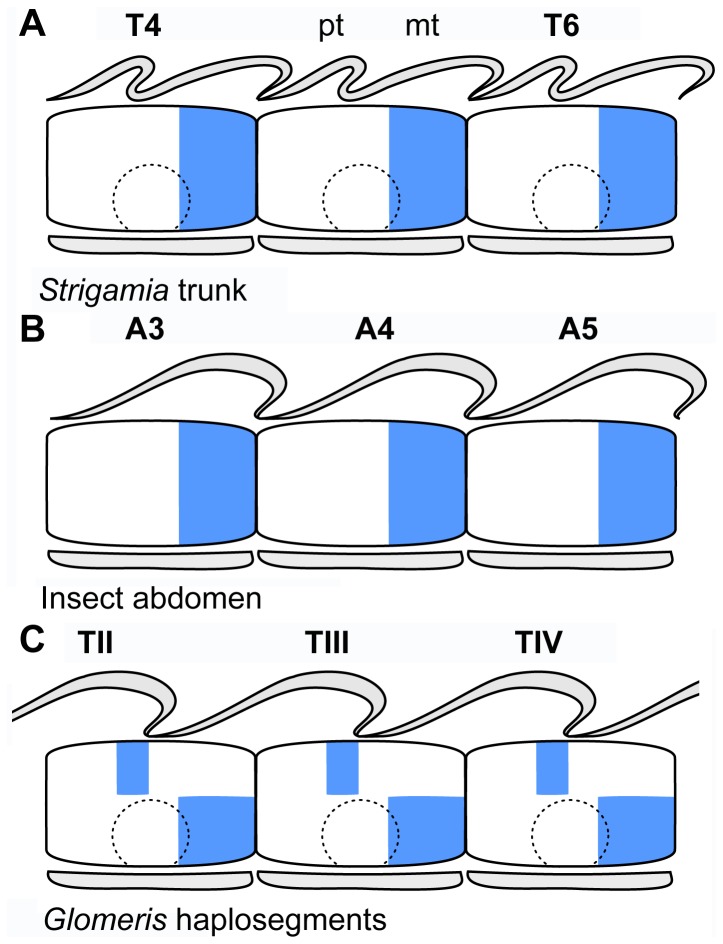
Schematic models of comparative dorsoventral expression of *engrailed* (blue), and correspondence with the tergite borders in extant arthropods. Anterior facing left. **A.** The expression of *engrailed* in the trunk segments of *Strigamia maritima* is reflective of the plesiomorphic condition of the arthropod trunk, consisting of a continuous dorsoventral stripe adjacent to the intersegmental boundary, and that extends into the limbs (*dotted line*). The posterior limit of the dorsal *engrailed* stripe is directly correlated with the posterior (meta)tergite (*mt*) border and the intersegmental boundary, and does not overlap with the anterior edge of the following (pro)tergite (*pt*). **B.** The expression of *engrailed* in the segments of the insect abdomen is similar to that observed in *Strigamia*, but differs in the absence of limbs [41], [47]–[50]. **C.** In the haplosegments of *Glomeris marginata,* the dorsal *engrailed* stripe is not expressed adjacent to the ventral intersegmental boundary, but rather anteriorly, approximately above the limbs; nevertheless, the tergite borders maintain the correlation with the expression of *engrailed* observed in *Strigamia* (A) and insects (B). The ventral side shows the typical activity of *engrailed* in the posterior portion of each segment [43], [51]. Other abbreviations: *Tn*, trunk tergite number *n*; *An*, abdominal tergite number *n*. Numbering in C follows the nomenclature used by Janssen et al. [43].

The results confirm previous findings on the early expression of *en* in embryos of *Strigamia* [55], [56], [58], and provide new information about the later differentiation along the mediolateral axis of the expression of this segment polarity gene, as well as its correlation with the position, elongation and dorsal closure of the trunk tergites (Fig. 8A). It is noteworthy that, despite the fact that the hemitergites initiate their formation as simple “lateral bumps” at stage 4, by stage 7 it is possible to discern the intrinsic differentiation between pretergite and metatergite due to the appearance of the intercalary furrow [54]. Additionally, the presence of the developing tracheal pits at stage 7 provides another important morphological reference point for following the differentiation of the dorsal and pleural sclerites that will constitute the mature exoskeleton. These cuticular features can also be contrasted with the dorsal expression of *en* in embryonic stages, and represent valuable landmarks for estimating the approximate expression domains of this gene in hatched individuals. Thus it is possible to extrapolate the expression domain of *en* in the postembryonic trunk segments, which would be largely consistent with the pattern observed in the embryonic stages (Fig. 8A). It is currently unknown whether *en* is active in the trunk of *Strigamia* for an unspecified amount of time following hatching, as it does in the lithobiomorph centipede *Lithobius peregrinus* [59], or if its expression ceases completely during late embryonic development after the formation of the last leg-bearing trunk segment at stage 8 [54]. The fact that *Strigamia* has an epimorphic development, unlike the anamorphic mode of *Lithobius*, could suggest that the segment patterning activity of *en* comes to a halt, or is otherwise too weak to be detected by in situ staining, once all the leg-bearing segments have formed prior to hatching. A possible exception would be the posterior extreme of the trunk, where the genital segment probably remains to be patterned (CB, unpublished data).

The observed dorsal expression of *en* in the trunk segments of *Strigamia* is coincident with approximately the posterior third of each tergite. More specifically, the posterior edge of each *en* stripe is directly correlated with that of the posterior (meta)tergite boundary of its corresponding segment, and does not overlap with the adjacent (pre)tergite (Figs 4D5; 8A). As mentioned earlier, a similar situation has been observed in the abdominal tergites of *Oncopeltus* [47] and *Drosophila* [41], [48], [49] (Fig. 8B); in the latter case, it is possible to readily distinguish between the posterior-most tessellated part of the *en*-positive intersegmental membrane, and the unpigmented, hairless acrotergite that defines the anterior-most part of the adjacent tergite. The observations made by Janssen et al. [43], [51] on the correlation of dorsal *en* stripes with the tergite borders in the trunk segments of *Glomeris* are in general agreement with the condition of the insect abdomen and the new data presented here, although there is a noticeable secondary offset relative to that on the ventral side (Fig. 8C). While the ventrolateral expression of *en* in *Glomeris* is restricted to the posterior region of each segment, as is typical for other arthropods, in the dorsal domain the situation is more complex. In the few anterior haplosegments (i.e. trunk segment bearing a single pair of legs [60]), the dorsal part of the stripes appears localized in the middle of each of the lobes that constitute the dorsal germ band, the so-called lateral plates of Dohle [43], [51]. Janssen et al. [43] considered these lobes as dorsal ‘segments’ in continuity with the ventral ones, which resulted in their interpretation of an anteriorly displaced *en* dorsal expression stripe relative to the ventral side; consequently, they concluded that the dorsal expression of *en* is probably independent of the plesiomorphic ventral region. There is no indication of the lineage of these populations of dorsal cells, and thus the precise continuity with the ventral segments is somewhat uncertain. Whatever their nature, however, it is clear from the data presented by Janssen et al. (see figs 1, 5 in [43]) that *en* is originally expressed in an almost continuous dorsoventral domain, although with different levels of intensity along the mediolateral axis. This observation is in accordance with the synchronous genetic and morphological patterning of the ventral and dorsal segmental units, even though the latter exhibits a considerable degree of secondary modification soon after their formation. We indeed think that the distinctive anterior displacement on the dorsal expression stripe of *en* observed in the haplosegments of *Glomeris*, could be simply the result of the degree of bending of the germ band due to the natural curvature of the developing embryos, as it is typical of many other genes in this arthropod [43], [51], [61]. In the posterior diplosegments (i.e. trunk segment bearing two pairs of legs [60]), however, the *en* stripes are in perfect continuity with the dorsal ones, even if there is not a one-to-one correspondence between dorsal and ventral segmental units. A noteworthy difference from the expression observed in the haplosegments, is that only every second *en* stripe actually extends to the laterodorsal tissues in this region, so that each dorsal stripe corresponds to two ventral ones; thus there is an alternating correspondence between the dorsal and ventral stripes of *en* (see fig. 5 in [43]). Regardless of these complications in dorsal *en* expression, there is still a direct correlation between the expression domain of this segment polarity gene and the formation of the tergite borders: in the haplosegments, the tergites are displaced anteriorly and thus cover the posterior half of one segment and the anterior half of the following one (i.e. haplotergite; [60]), whilst in the diplosegments each tergite covers two ‘ventral’ segments (i.e. diplotergite; [60]), exactly mirroring the dorsal pattern of *en* ectoderm expression [43], [51].

The close correspondence between the dorsal expression of *en* and the formation of the tergites in the trunk segments of phylogenetically distant representatives of Hexapoda (*Oncopeltus*, *Drosophila*) and Myriapoda (*Strigamia*, *Glomeris*) is indicative that this relationship represents a highly conserved, and almost certainly ancestral, patterning mechanism of the ectoderm derivates in Mandibulata. Amongst Chelicerata, detailed information on the expression of segment polarity genes is only available from a few araneaeid species. However, the pattern of hemitergite formation and dorsal closure in the opisthosoma of the cobweb spider *Parasteatoda tepidariorum* [62] seems to be broadly comparable with the dorsoventral expression stripes of *en* observed in the posterior region of the orb weaving spider *Cupiennius salei* [42]. These observations strongly suggest that the correlation between *en* expression and tergite formation represents the symplesiomorphic condition for the development of the laterodorsal exoskeletal elements in crown-group Euarthropoda.

### Implications for the Segmental Organization of the Trilobite Trunk

#### Trilobite dorsoventral exoskeletal organization

**Figure 9:**
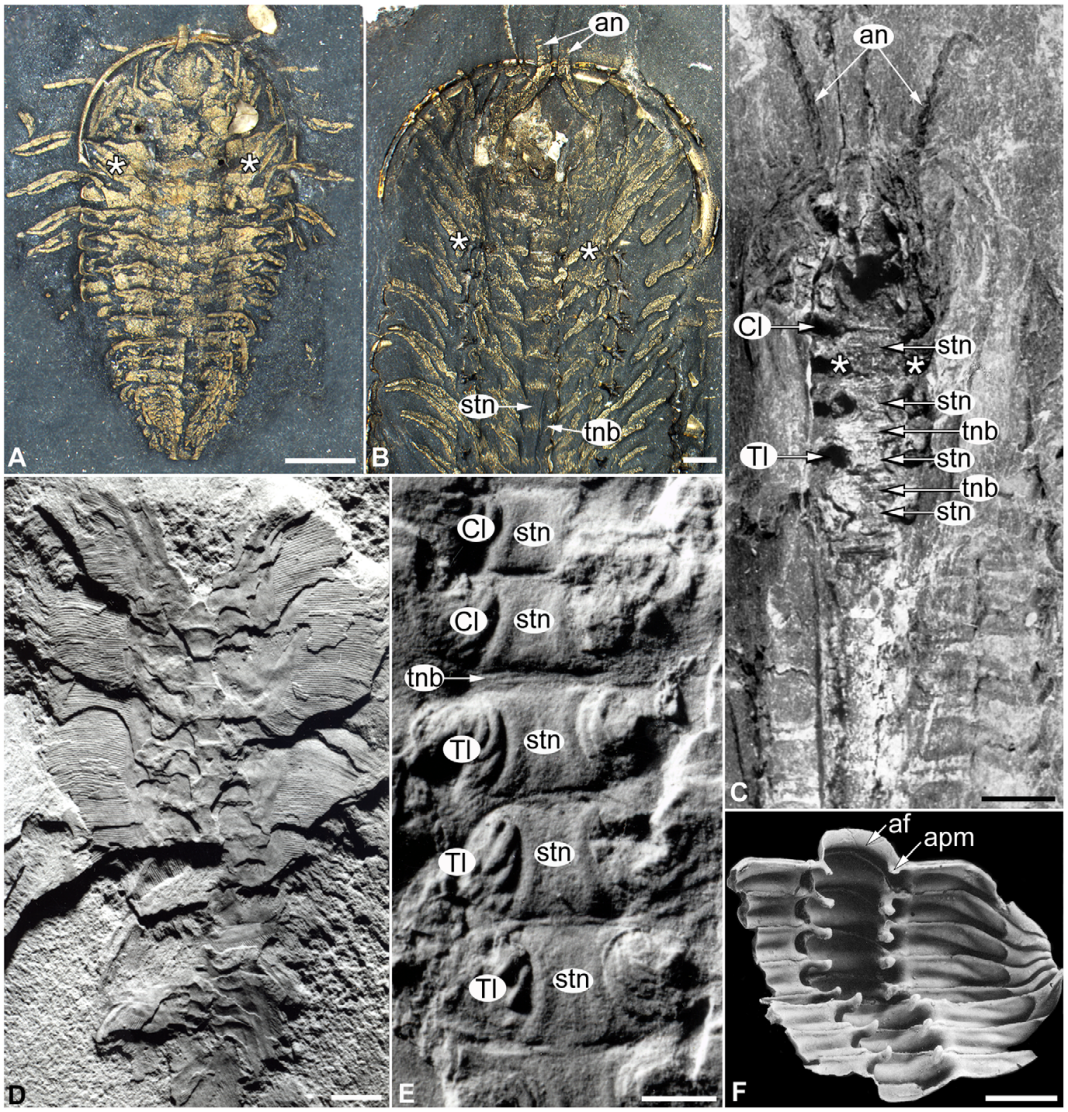
Trilobites and trilobite-like arthropods with exceptionally preserved dorsoventral exoskeletal elements. Anterior facing up. **A.** Dorsal view of pyritized specimen of the olenid *Triarthrus eatoni* (USNM 400935) from the Late Ordovician Beecher’s trilobite bed (USA), showing typical trilobite dorsal morphology and organization of the trilobite-like articulation. A pair of walking legs (*asterisk*) is located under the cephalo-thoracic articulation. **B**. Unmineralized ventral exoskeletal morphology of *Triarthrus eatoni* (USNM 400943), including the sternite series (*stn*) connected by intersegmental tendinous bars (*tnb*). **C.** Dorsal view of ventral internal mould of the phacopid *Placoparia cambriensis* (NMW 91.46G) from the Ordovician of Wales (UK), showing three-dimensional preservation of the sternite series, transverse tendinous bars, and sites for cephalic (*Cl*) and thoracic (*Tl*) walking leg attachment (original film courtesy of the Whittington archives, University of Cambridge). **D.** Exceptionally preserved ventral morphology of the lightly sclerotized trilobite-like nektaspidid *Misszhouia longicaudata* (ELRC 11555) from the Lower Cambrian Chengjiang lagerstätte (south China) (original print courtesy of GD Edgecombe). **E.** Ventral view of sternite series of *Misszhouia longicaudata* (ELRC 11590b) showing the tendinous bars and sites for walking leg attachment in detail (original print courtesy of GD Edgecombe). **F.** Ventral view of an incomplete trunk of the phacopid *Geesops sparsinodosus* from the Middle Devonian of Eifel (Germany) (reproduced from [33], with permission of the Palaeontological Association), showing the sites for muscle attachment on the visceral side of the tergite series, and morphological continuity between the articulating furrow (*af*) and the apodemes (*apm*). Scale bars: 1 mm for A; 2 mm for B–D; 1 mm for E; 0.5 mm for F.

**Figure 10:**
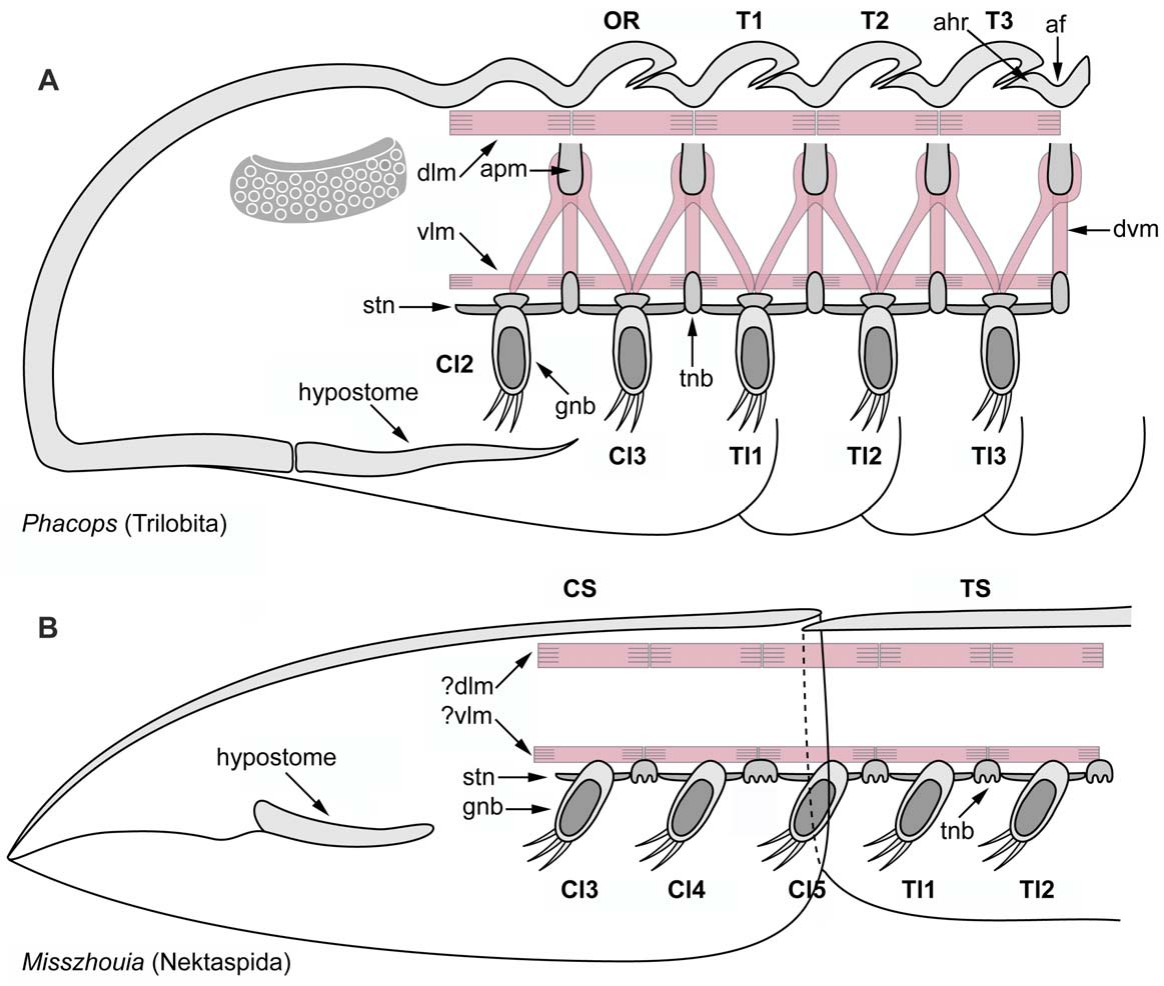
Reconstructions of the dorsoventral morphology of trilobites and trilobite-like arthropods. Anterior to the left. **A.** Exsagittal longitudinal section of the phacopid *Phacops* showing typical trilobite exoskeletal organization [20], [27], [32], [33]. The biomineralized tergites (*Tn*) overlay the lightly sclerotized series of sternites (*stn*) that are connected by flexible tendinous bars (*tnb*). Each sternite bears a pair of laterally attached gnathobasic (*gnb*) walking legs. The reconstruction of the dorsal (*dlm*) and ventral (*vlm*) longitudinal muscles follow the functional requirements for typical arthropod locomotion, and are shown attached to specific regions of the visceral exoskeleton such as the articulating furrow (*af*) and the apodemes (*apm*); note that although the apodemes actually are in direct contact with the visceral side of the tergite at the level of the articulating furrow (see Fig. 8f), such connection is not shown in here due to the schematic nature of this representation. The trilobite-like articulation consists of the anteriorly shifted position of the tergite borders relative to the sternite borders; consequently, a pair of cephalic legs (*Cln*) is located under the cephalo-thoracic articulation, and the thoracic legs (*Tln*) under each tergite-to-tergite junction. **B.** Longitudinal section of the nektaspidid *Misszhouia longicaudata* showing exoskeletal organization in an unmineralized trilobite-like arthropod [13]. The tergites of nektaspidids are fused into a thoracopygidial shield (*TS*), with a single articulation at the cephalo-thoracic junction; as diagnostic for the trilobite-like articulation, a pair of cephalic legs is positioned directly under this region. Aspects of the ventral morphology, such as the sternite series, tendinous bars and limb attachment sites, are very similar to those of trilobites; however, there is no clear indication for muscle attachment sites on the visceral side of the thoracopygidial shield. Other abbreviations: *OR*, occipital ring; *ahr*, articulating half ring; *dvm*, dorsoventral muscle; *CS*, cephalic shield.

**Figure 11:**
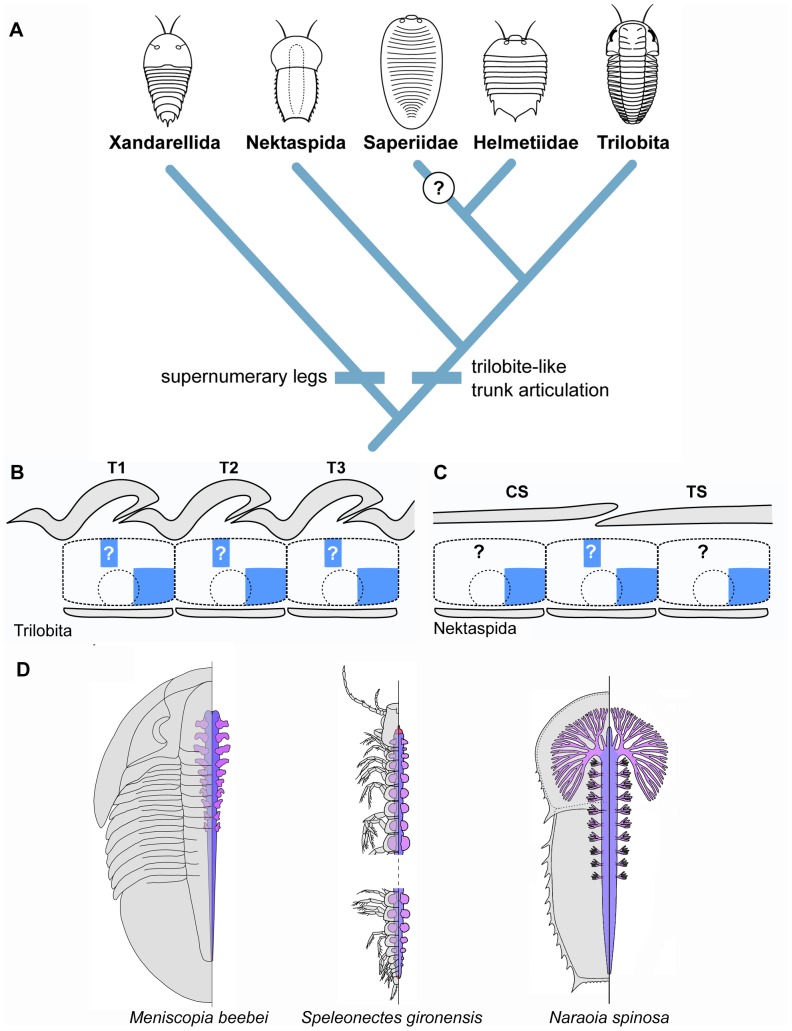
Phylogenetic relationships, trunk segmentation, and gut metamerism of trilobites and their close relatives. **A.** Phylogenetic position trilobites relative to other various groups of trilobite-like arthropods [68]. The trilobite-like articulation is synapomorphic for a distinct clade that includes trilobites, helmetiids and nektaspidids; the presence of this condition in saperiids is uncertain due to preservation and rarity of specimens. Xandarellids do not feature this type of exoskeletal organization, but rather are distinguished by the presence of several pair of walking legs under the posterior tergites (i.e. polypody). **B.** Inferred dorsoventral expression of *engrailed* (blue) in the trunk of trilobites and nektaspidids. The ventral expression of *engrailed* was most likely typical for arthropods in general, restricted to the posterior part of the segment, adjacent to the intersegmental border, and extending into the posterior portion of the limbs. The anteriorly shifted tergites that conform the trilobite-like articulation, coupled with the ancestral correlation between *engrailed* and the position of the tergites, strongly suggest that the domain of this gene was also anteriorly shifted, and thus expressed dorsally relative to the limbs. In nektaspidids, it is only possible to make accurate inferences on the dorsal expression of *engrailed* on the cephalothoracic articulation; consequently, the dorsal activity of this gene on the rest of the trunk segments remains uncertain. **C.** Metamerism of the digestive tract in trilobites, trilobite-like arthropods and remipedes (modified from [72]). The digestive tract of the ptychopariid *Meniscopia beebei* shows a metameric organization that consists on several paired gut diverticulae that are located exactly under the tergite-to-tergite articulation throughout the trunk. The remipede *Speleonectes gironensis* also features paired gut diverticulae; however, in this case the diverticulae are confined within each segment, indicating that they follow the plesiomorphic ventral pattern of arthropod segmentation. The diverticulae in the gut of the nektaspidid *Naraoia spinosa* is consistent with the organization observed in *Meniscopia*, even though the only functional articulation.

Most aspects of the palaeobiology of trilobites are exclusively known from the morphology of their biomineralized dorsal exoskeletons. There are little more than 20 trilobite species from which the exceptionally preserved ventral tissues have been described, covering a considerable range of ages, taxonomic groups and preservation styles (Fig. 9) [1]. Although they represent a modest sample out of the more than 19,000 [63] trilobite species reported to date, these taxa provide crucial information about their soft ventral anatomy, and also give a clear indication that most trilobites had a remarkably consistent body organization throughout their evolutionary history (Figs 9, 10). The exoskeletal constituents of the trilobite trunk include the aforementioned biomineralized tergites on the dorsal side, which overlay a series of lightly sclerotized hourglass shaped sternites on the ventral side that were interconnected by flexible tendon-like transverse structures (i.e. tendinous bars), most likely homologous to the intersegmental membrane of extant arthropods (Figs 9B–E) [27]. Each sternite is bordered by a pair of biramous gnathobasic walking legs that insert laterally with respect to the longitudinal axis of the body (Figs 9, 10). While the basic dorsoventral components of the trilobite exoskeleton do not differ significantly from those typical of arthropods in general, the relative disposition of some of these elements is indicative of a derived type of organization (Fig. 10). Edgecombe and Ramsköld [13] drew attention to the discussions on the precise number of limb pairs found within the trilobite head. Depending on the author, this number fluctuates between one pair of antennae and three [25], [26] or four [29], [31] pairs of walking legs. Through an extensive examination of exceptionally preserved trilobites and other closely related non-trilobite arthropods (e.g. nektaspidids, helmetiids) (Figs 9D, E; 10), Edgecombe and Ramsköld [13] highlighted that part of the problem in determining the precise number of walking legs in the head stems from the fact that one pair of limbs, usually the fourth, is positioned directly under the cephalo-thoracic articulation (Fig. 9A–C). Different trilobite (e.g. *Phacops* [37]) and trilobite-like (e.g. *Misszhouia* [64], [65]) species may deviate from the four-legged condition found in the head of most trilobites (see Table 1 in [1]), but in all cases the presence of a distinct “trilobite-like articulation” still applies (Fig. 10). As a consequence of this organization, the corresponding tergite and sternite borders are not vertically aligned, but rather out of phase by approximately half a unit relative to each other. This peculiar condition is not exclusive to the cephalo-thoracic boundary, but represents the standard exoskeletal organization throughout the whole length of the trunk. More recent studies have confirmed the widespread distribution of this character among trilobites and their close relatives [33], [66]–[68], and thus it can be readily considered as a fundamental aspect of the body organization of these extinct arthropods (Figs 10, 11A; see discussion on agnostid “trilobites” below).

It is very unlikely that the peculiar trunk organization of trilobites is merely the result a taphonomic artefact caused by the decay of the internal tissues or compression after burial. Specimens with soft tissue preservation of *Kiisotoria saperi*, an Early Cambrian non-trilobite arthropod from Greenland, show the presence of transverse ligament-like structures comparable to the tendinous bars of trilobites [69]. Although originally interpreted as dorsal tendons [69], these features are remarkably regular in their appearance and clearly restricted to the axial region of the trunk, characteristics which are not comparable with those of muscular or connective tissues preserved in other arthropods from the Sirius Passet Lagërstatte [67], [70]. This suggests that these transverse structures likely represent the cuticular remains of the lightly sclerotized ventral side of the animal, which are in almost perfect correspondence with the tergite margins in the dorsal side. These observations indicate that exceptionally preserved fossil material can faithfully replicate the original organization of the trunk, matched or mismatched, given the presence of ventral and dorsal exoskeletal remains.

Edgecombe and Ramsköld [13] commented on the segmental organization of trilobites and other closely related forms: *“We will not enter into the detailed discussion on whether the tergite boundaries in these arthropods coincide with (segment) boundaries. Arbitrarily assuming that they do, the (segments) … must be obliquely inclined, with the ventral part more anterior than the dorsal”*. Although the formation and maintenance of the body segments in arthropods is a complex process that requires a precise patterning of the ectodermal and mesodermal derivates, the interpretation for obliquely inclined segments in trilobites and their close relatives would imply a considerable morphogenetic rearrangement of the former. Instead, the plesiomorphic correlation between the position of the tergite borders and the expression of *en* in extant arthropods can provide an alternative explanation for this exoskeletal organization. The highly conserved ventral segmentation patterning and associated expression of *en* within Panarthropoda (Fig. 1C) strongly suggests that the expression domain of this segment polarity gene in the trilobite trunk did not differ significantly from the status quo; i.e. the ventral stripe would have been active on the posterior third of each segment, as indicated by the position of the exceptionally preserved sternites, reaching into the posterior portion of each corresponding walking leg (Figs 11B, C). The dorsal side, however, represents a slightly different scenario. Based on the correlation between *en* and the development of the mature tergite borders observed through all the morphogenetic stages in *Strigamia* (this study), insects [41], [47]–[49] and *Glomeris* [43], [51], it can be inferred that the dorsal expression domain of this gene did not match that of the ventral side in trilobites, but rather was displaced anteriorly relative to the former following the disposition of the exoskeletal elements that conform the trilobite-like articulation (Fig. 11B). The dorsal *en* stripe in the trilobite trunk would not have been located in the posterior region of the segment, but rather approximately in the middle of it, just above the site of limb attachment to the body. The same interpretation is also applicable for trilobite-like taxa in which most of the tergite boundaries have become fused into a single trunk (or thoracopygidial) shield. In the case of nektaspidids, for example, the only functional articulation is found at the cephalo-thoracic boundary (Figs 10B, 11C); however, the characteristic organization of the trilobite-like articulation in these bizarre arthropods is indicative of a similar type of dorsoventral mismatch (Figs 11A, C), even if it is not possible to address the expression of *en* in other parts on the dorsal side of the body due to a lack of suitable morphological proxies. The inferred domains of dorsal en expression in the trunk of trilobites and trilobite-like arthropods are coincident with the tergite junctions, which in all likelihood were interconnected in life by a very flexible, unsclerotized intertergal membrane. This correlation inevitably draws parallel to the interpretations made by Jacobs et al. [71] on the fundamental involvement of *en* in the process of invertebrate skeletogenesis, acting as a localized suppressor of heavy sclerotization and biomineralization in the ectoderm, and defining the boundaries of the exoskeletal elements. Within this context, the presence of dorsoventral differences in the expression domains of *en* in trilobites and their close relatives not only gains additional support, but also becomes a basic prerequisite in terms of functional morphology given the distinct arrangement of their exoskeleton.

#### Additional insights from gut metamerism

The discussion so far has dealt exclusively with ectodermal derivates; however, the information available on the internal anatomy of trilobites also contributes to the interpretation of trilobite trunk segmentation presented here. Lerosey-Aubril et al. [72] recently provided a detailed account of the phosphatised guts in some Cambrian representatives. The digestive tract of some trilobites consists of a relatively simple gut that bears several pairs of diverticulae on its anterior half (Fig. 11D). The exceptional preservation of these digestive structures allows a precise comparison with the disposition of the adjacent dorsal exoskeletal elements, and it is clear that each pair of gut diverticulae are located exactly under the tergite-to-tergite junction. Viewed from a ventral perspective, this position corresponds to the middle of each segment; thus it seems that the digestive system of trilobites was patterned following the ancestral ventral segmental organization of the body (Fig. 11B) unlike the derived disposition of the dorsal exoskeletal elements. These observations are corroborated when comparing the position of the trilobite gut diverticulae with those of the remipede *Speleonectes gironensis* [72]; here, these structures are clearly confined within the boundaries of their corresponding sternites and tergites, which is reflective of the plesiomorphic condition of the arthropod segments (Fig. 11D). The same argument can be made for non-trilobite arthropods (e.g. nektaspidids) in which the gut diverticulae are also preserved exactly in the same position under the cephalo-thoracic articulation, and can be traced further back posteriorly indicating the position of some of additional trunk segments (Figs 10B, 11D) [64], [65].

#### Muscle patterning and segmentation

Hessler [20] discussed in some detail the evidence for muscle attachment sites in the trilobite trunk as an argument in favour of the interpretation of these structures as indicators of the primordial myosegmental organization [21], and concluded that the longitudinal dorsal muscles must have been anchored to the visceral side of the articulating furrow in each tergite (Figs 1A1; 9F; 10A). The latter view is essentially universally accepted, as the articulating furrow represents the part of the tergite that lies in closest proximity with the body wall, and is also associated with the lateral apodemes of the trunk that served as attachment sites for the extrinsic muscles connecting the legs with the body (Figs 9F; 10A) [27], [32], [33], [37]. Given the conspicuous segmental organization of the dorsal longitudinal muscles and their relationship with the cuticular structures in extant arthropods [18], [73], [74], as well as the activity of specific genes expressed at the intersegmental borders that are essential for muscular patterning in insect larvae [75], [76], it would seem reasonable to view the longitudinal trunk musculature as indicative of the segmental organization in trilobites. Although the former observations are true for some arthropods and/or during certain ontogenetic stages [74], this does not apply to all extant representatives. It has been shown that the attachment site of the dorsal longitudinal muscles changes during post-embryonic ontogenetic development in the abdomen of holometabolous insects. This is particularly clear in *Drosophila*, where the larval dorsal longitudinal muscles follow a clear intrasegmental organization, whilst the functionally equivalent adult musculature has a shifted intersegmental position [50], [73], [77].

Although it is well known that several trilobite species underwent considerable changes in their dorsal exoskeletal morphology during their early ontogenetic growth [19], it is currently impossible to make an objective assessment of the impact of these changes on the organization of the musculature relative to its attachment sites, as well as the relationship of these with the segmentation pattern of the trunk. Given this uncertainty, we consider that the position of the intersegmental boundaries in the trilobite trunk inferred from the plesiomorphic pattern of panarthropod ventral segmentation, and the anteriorly shifted tergites associated with the derived expression domain of *en* (Fig. 11B), represent more accurate indicators of the segmental patterning of this tagma than the exoskeletal sites of muscle attachment. The resulting interpretation is in general agreement with the hypotheses that postulate an indirect correspondence between the dorsal exoskeletal elements and the underlying segments [12], [19], [20] (Fig. 1B). Unlike the former models, however, this view no longer relies on extrapolations from the now defunct notion of secondary segmentation [20], [21], nor does it dwell on speculative interpretations about the posterior segmental organization of the controversial trilobite head shield [19], thus providing a more satisfactory argument within the context of the contemporary understanding of extant arthropod segmentation and development.

#### Evolutionary significance of dorsoventral mismatch

The proposed dorsoventral mismatch of segment polarity gene expression in the trilobite trunk clearly deviates from the plesiomorphic arthropod condition consisting of a dorsoventrally continuous stripe of *en* expression (e.g. *Strigamia*, Fig. 8A), but is strikingly reminiscent of the expression pattern of *en* observed in the haplosegments of *Glomeris* [43], [51] (see discussion above) (Fig. 8C). This comparison carries significant implications for understanding the developmental patterning of the trilobite trunk, as well as those of other extinct Palaeozoic arthropods, as the genetic processes responsible for the peculiar segmentation of this diplopod have been the subjects of considerable scrutiny in recent years. Apart from the anteriorly displaced expression of *en* in the anterior dorsal segmental units of *Glomeris*, Janssen et al. [51] report that, unlike the ventral side, this region does not seem to express *wingless* (*wg*), a segment polarity gene that is normally active immediately anterior to the expression stripe of *en* [45], [49], together establishing the anteroposterior axial identity of each segment. To account for this observation, Janssen et al. [51] hypothesized that the *wg*-like anterior patterning signal could possibly be substituted by the interaction of *hedgehog*, a segment polarity gene with a similar expression domain to that of *en*, and a number of additional genes (*optomotor-blind*, *cubitus-interruptus, decapentaplegic*) that are also expressed in the dorsal segmental units of *Glomeris*. This proposed mechanism is largely based on analogy to a process that has been reported in the abdominal ventral pleurae [49], [78] and wing disc [79] of *Drosophila*, which are characterized by similar patterns of gene and morphogen activity.

**Figure 12:**
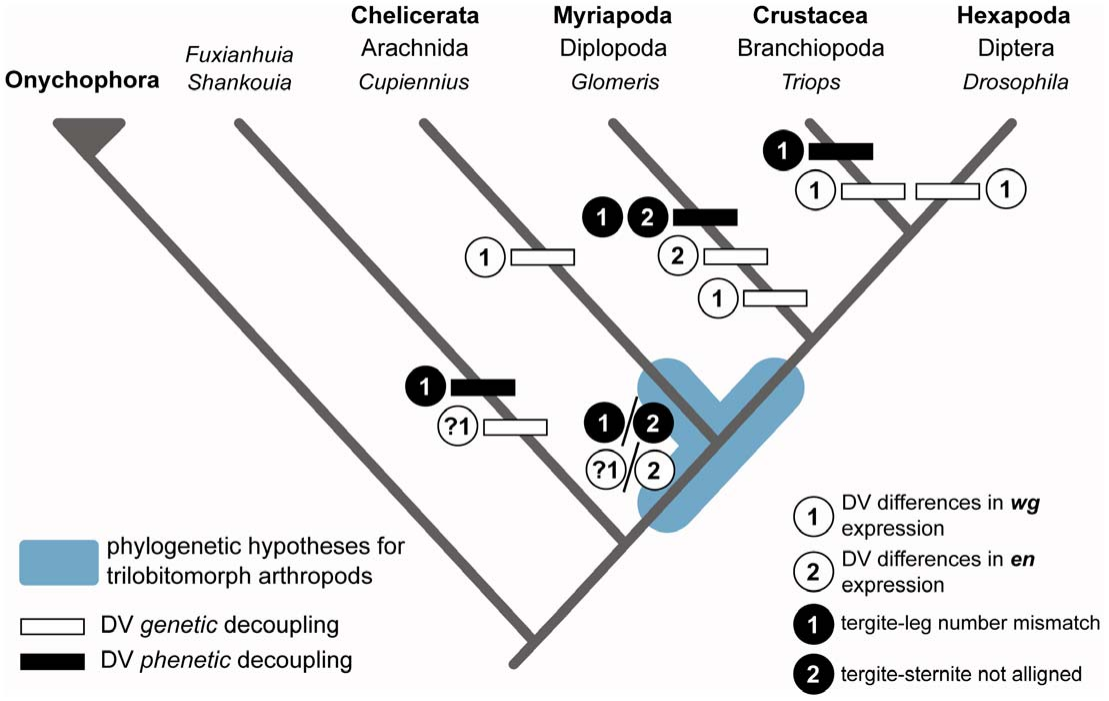
Phylogenetic distribution of dorsoventral trunk segmental mismatch within total-group Euarthropoda; note that these represent peculiar cases, and thus are not necessarily reflective of the fundamental organization of each major arthropod group as a whole. Among extant representatives, dorsoventral segmental mismatch, acting at the genetic and/or phenetic levels, has been reported in the trunk of *Drosophila* [49], [78], [79], *Triops* [80], and *Glomeris* [43], [51]. In *Cupiennius*, the dorsoventral mismatch is observable only at the gene expression level [42]. In most cases, differences in dorsoventral expression of segmentation genes are correlated with morphological segmental mismatch. In extinct taxa, various types of dorsoventral segmental mismatch are present in fuxianhuiids [31], [53], trilobites and trilobite-like arthropods, but the extent of a possible genetic mismatch is uncertain in most cases (see discussion in text).

It is of course impossible to extrapolate whether the dorsoventral decoupling of the exoskeleton in the trilobite trunk followed a similar genetic differentiation as the one proposed for the haplosegments of *Glomeris* [43], [51], or whether the dorsoventral mismatch might simply be the result of the morphogenetic forward shift of the dorsal tissue. A sensible conclusion, in any case, would be to consider that the similarity between the trunk of trilobites (and other closely related taxa) and the haplosegments of *Glomeris* could be the result of evolutionary convergence that would have at least one parallel at the level of gene expression (i.e. anteriorly displaced *en* dorsal stripe), which consequently results in a close morphological analogue (i.e. dorsoventral exoskeletal mismatch). Indeed, among extinct and extant arthropods, genetic and phenetic differences in the dorsoventral segmental patterning of the trunk region can be found in several additional cases (Fig. 12). In *Drosophila*, for instance, there are a number of segmentation genes that are dorsoventrally differentially expressed or regulated, both under natural conditions and induced experimentally (see discussion in [43]). The spider *Cupiennius salei* offers another example, as *wg* (i.e. *Cs-wg*) is not expressed in the most ventral region of the germ band; however, it may be possible that the widespread dorsoventral expression of the gene *Cs-Wnt5-1* is responsible for establishing the anteroposterior polarity of the germ band [42]. Conversely, *wg* is apparently not expressed on the dorsal side of the notostracan *Triops longicaudatus* during post-embryonic segmentation (*Tlwnt-1* [80]). The case of *Triops* is a particularly interesting one: apart from featuring a derived type of dorsal patterning at the genetic level (i.e. lack of dorsal *Tlwnt-1* expression), it has an additional morphological parallel with the diplosegments of *Glomeris* in that the posterior tergites are associated with more than one pair of legs (i.e. polypody [81]). This represents a rather different case of non-correspondence between ventral and dorsal segmentation, in which the tergite borders correspond to the plesiomorphic ventral pattern of segmentation, but part of the latter does not have a corresponding dorsal structure.

This consideration can be further applied to extinct taxa that are also characterized by the presence of supernumerary pairs of limbs per tergite on the posterior region of the trunk. Among trilobite species with exceptional limb preservation, there are some confirmed cases in which there are several pairs of walking legs clustered in the posterior portion of the trunk, the so-called pygidium [1]. The most notable example of this condition can be found in the Late Ordovician olenid *Triarthrus eatoni* (Figs 9A, B) (see figs 3, 4 in [30]). Here, the dorsal exoskeletal morphology of the adult pygidium consists of five axial rings, which following the organization of the trunk should only correspond to five pairs of walking legs on the ventral side; however, it is possible to observe at least 10 pairs of limbs associated with this body region. Within the trilobite-like arthropods, xandarellids represent the only confirmed case of polypody (Fig. 11A), as the posteriormost tergites of some representatives (e.g. *Cindarella eucalla, Xandarella spectaculum*) are associated with several pair of legs, unlike those that form anterior part of the trunk that only bear a single leg pair [31], [82]. It is noteworthy that xandarellids do not feature the distinctive trilobite-like articulation [13], [68] (Fig. 11A), and thus it is possible to recognize a significant diversity in terms of segmental mismatch in trilobites and trilobite-like arthropods, which can be independently expressed both in terms of an anterior shift of the dorsal exoskeleton, and/or in the presence of supernumerary pairs of walking legs per tergite (Fig. 12). Polypody has also been reported in fuxianhuiids (e.g. *Fuxianhuia protensa*, *Shankouia shenghei*), a controversial lower Cambrian group of rather primitive-looking arthropods that have been commonly interpreted as basal members of the euarthropod stem lineage [24], [31], [53], [82]. The ubiquitous presence of supernumerary limbs, and by implication the genetic patterning mechanism responsible for their architecture, in these allegedly primitive arthropods can be explained in two ways: either the dorsoventral decouplement of segmentation represents an ancestral developmental trait [43] that could potentially form part of the ground pattern of total-group Euarthropoda, or this complex organization is a derived feature that was acquired in parallel within the fuxianhuiid branch, among several others (Fig. 12). Taking into consideration the plasticity exhibited by extant representatives in terms of the dorsoventral mismatch diversity across different lineages, and the fact that this represents an apparently derived trait within the phylogenetic context of most of the major extant groups, it seems most likely that fuxianhuiids and other extinct taxa developed the polypodous condition independently from each other (i.e. convergence). Ultimately, the fossil evidence indicates that the fundamental genetic components and processes responsible for orchestrating the observed plethora of dorsoventral mismatched trunk segmentation phenotypes were already part of an intrinsic arthropod developmental toolkit that can be traced back to some of the oldest known representatives of this phylum (see also discussion in [51]). This conclusion is in accordance with recent molecular clock calibrations that estimate an Ediacaran age (630-542 Ma) for the early diversification of total-group Euarthropoda [83], and thus is suggestive that the first instances of derived modes of trunk segmentation developed remarkably early in the evolutionary history of the group. Given the broad phylogenetic distribution of the examples described above (Fig. 12), it is possible that the reiterated and independent appearance of trunk dorsoventral segmental mismatch in different arthropod lineages is the result of an homocratic process, i.e. shared patterns of regulatory gene expression between organs in different taxa [43], [84], [85]; however, great caution must be exercised with this interpretation, as the concept of homocracy explicitly applies to those morphological features that share the expression of the same patterning genes, and thus its unambiguous recognition in extinct taxa is beyond the prowess of the arthropod fossil record.

#### Phylogenetic considerations for agnostid “trilobites”

The synapomorphic dorsoventral segmental mismatch in the trunk of trilobites, and some of their close relatives, has implications for the phylogenetic position of agnostids, a group of diminutive arthropods that have been traditionally regarded as trilobites but whose precise affinities are contentious [63], [86]–[88]. Detailed information on the ventral morphology of agnostids is only available from *Agnostus pisiformis*, from the Upper Cambrian of Sweden [86]. The incredible morphological detail preserved in this species clearly indicates that the trunk organization does not feature the distinctive trilobite-like articulation (Fig. 10). Instead, the relationship of the dorsal and ventral exoskeletal components, as well as the position of the trunk appendages, is reflective of the plesiomorphic arthropod condition as recognized here (Fig. 8A). Agnostids closely resemble eodiscid trilobites in overall appearance and size of the dorsal exoskeleton, which has been traditionally utilized to justify the trilobite interpretation of the former (see discussion in [87]). However, the trunk appendage structure of *Agnostus pisiformis* drastically differs from that recognized in polymeroid (i.e. non-agnostid) trilobites with soft-tissue preservation (Fig. 9A), but instead is reminiscent of stem-crustacean limbs [86]–[88].

Within the context of our findings on trilobite segmentation, it is possible to provide two alternative interpretations for the affinities and trunk organization of *Agnostus pisiformis*: if this species is a bona fide trilobite, then its pattern of trunk segmentation suggests the effect of an atavistic process (i.e. regression to the ancestral state) that could be associated with the reduced body size that is characteristic of agnostids; alternatively, the trunk organization may provide indication that the position of agnostids lies outside Trilobita, and probably within the stem-lineage of Crustacea or even Mandibulata, but have acquired a similar dorsal morphology with eodiscid trilobites convergently. Haug et al. [88] noted that the mode of ontogenetic growth that characterizes polymeroid trilobites and agnostids, consisting in the differentiation of new segments before the appearance of the corresponding appendages that develop from a posterior growth zone, is also found in some stem- and crown-group crustaceans. Based on the plesiomorphic pattern of trunk segmentation observed in *Agnostus pisiformis*, coupled with the fact that the mode of trunk development in polymeroid trilobites and agnostids most likely represents an ancestral trait of total-group Mandibulata [88], we consider that the available evidence favours the interpretation of non-trilobite affinities for agnostids. However, we acknowledge that this information is still insufficient to precisely pinpoint the phylogenetic position of this problematic clade within total-group Euarthropoda.

### Conclusions

The difficulties associated with resolving the correlation, or lack thereof, of the segments that comprise the trilobite body with their respective exoskeletal elements stems from the fact that the morphological information available from the fossil record is inevitably incomplete, and thus poses unique challenges to the study of development and segmentation in extinct arthropods. Through the analysis of embryonic and postembryonic stages of *Strigamia*, it has been possible to corroborate the link between the development of the tergites and the associated dorsal expression of the segment polarity gene *en*, a relationship that can be readily considered as symplesiomorphic for Euarthropoda. The fact that this correlation is ubiquitous and persistent, even in extant representatives in which the trunk segmentation is clearly modified relative to the ancestral arthropod condition, enables to make precise inferences about the expression domains of *en* in the trunk of exceptionally preserved trilobites. This information leads to the conclusion that the segment and tergite borders of trilobites, as well as some of their close relatives, were not perfectly aligned with each other, which is indicative of a derived and widespread type of dorsoventral segmental mismatch in this diverse and early arthropod group. The interpretations on the segmental patterning of the trilobite trunk drawn from the ectodermal derivates of these arthropods are corroborated by additional information on further aspects of the exceptionally preserved internal anatomy, such as the structure of the digestive tract. Conversely, the metameric arrangement of the longitudinal musculature is deemed inadequate to address the segmental organization of the trilobite trunk due to the effect of potential postembryonic morphogenetic movements during ontogeny. These findings carry wider evolutionary implications for understanding the processes of arthropod segmentation in some of the oldest representatives of the group, and suggest the reiterated occurrence of a derived type of dorsal gene expression (i.e. anteriorly displaced *en* stripe) that is ultimately responsible for the morphological pattern of trunk segmental mismatch in several disparate groups of extinct (trilobites and closely related taxa) and extant (e.g. haplosegments *Glomeris*) arthropod throughout their long and successful evolutionary history.
